# Brief screening items to identify spanish-speaking adults with limited health literacy and numeracy skills

**DOI:** 10.1186/s12913-015-1046-2

**Published:** 2015-09-14

**Authors:** Rashmi Singh, Laura Scott Coyne, Lorraine S. Wallace

**Affiliations:** The Ohio State University, Wexner College of Medicine, Columbus, OH 43210 USA; University of Kentucky, Lexington, KY 40506 USA; Department of Family Medicine, The Ohio State University, 2231 North High Street, Columbus, OH 43210 USA

**Keywords:** Health literacy, Numeracy, Spanish-speaking, Validation studies

## Abstract

**Background:**

Limited health literacy (HL) and numeracy have been shown to be associated with a wide array of poor health-related outcomes, knowledge, and behaviors. The purpose of this study was to evaluate the clinical utility of brief HL and numeracy screening items in identifying Spanish-speaking adults’ HL and numeracy skills.

**Methods:**

We studied convenience samples of native Spanish-speaking adults in Columbus, Ohio. A trained research assistant administered sociodemographic items, HL and numeracy screening items, Short Assessment of Health Literacy (SAHL), and Newest Vital Sign (NVS) to participants in Spanish.

**Results:**

Participants (*n* = 151) averaged 36.8 ± 11.0 years of age and 54.7 % were female. Average SAHL score was 15.7 ± 2.8 (range = 4 to 18), while the average NVS score was 1.7 ± 1.5 (range = 0 to 6). “How confident are you filling out medical forms by yourself?” performed best in detecting limited NVS scores (AUROC = 0.66; 95 % confidence interval [CI] = 0.57–0.75), limited/marginal NVS scores (AUROC = 0.75; 95 % *CI* = 0.65–0.84), and inadequate SAHL scores (AUROC = 0.69; 95 % CI = 0.58–0.79).

**Conclusion:**

A single HL screening item is useful for quickly estimating HL and numeracy skills in native Spanish-speaking adults.

## Background

Hispanics are one of the fastest-growing minority groups in the United States, as well as a group considered to be at particularly high risk for problems associated with low health literacy (HL) and numeracy [1]. A growing body of literature documents the strong association of limited HL and numeracy with a wide array of poor health-related outcomes, knowledge, and behaviors [2]. Among Spanish-speaking adults in the United States specifically, those with limited HL and/or numeracy have shown to have difficulty understanding and acting upon emergency department discharge instructions [3], are less likely to adequately dose pediatric medications correctly [4], are at increased risk of seeking care at emergency departments and urgent care centers in response to their children’s asthma exacerbations [5], and are less likely to receive routine mammography [6].

While valid and reliable HL assessment tools such as the Rapid Estimate of Adult Literacy in Medicine [7], Test of Functional Health Literacy in Adults [8], Health Literacy Skills Instrument [9], Health Literacy Assessment using Talking Touchscreen Technology (Health LiTT) [10], and Health Literacy Management Tool (HeLMS) [11] are available for predominantly research purposes, time constraints often limited their use in busy clinical settings. Recognizing that it is often difficult for clinicians to identify patients at risk of limited HL based the conversation exchange during clinical encounters [12–14], shorter HL assessment tools such as the Short Assessment of Health Literacy (SAHL) [15], Newest Vital Sign (NVS) [16], and Communicative, Functional, Critical Health Literacy scales [17, 18] could, potentially, be useful in busy clinical settings to gauge a patient’s HL skills. The NVS—which can be administered in less than three minutes [19, 20]—is perhaps one of the most feasible HL assessment tools for use in routine clinical practice.

Among English-speaking populations, studies have revealed mixed results regarding whether patients’ feel comfortable or not disclosing their limited HL to health care providers [21–24]. For instance, in studies were the REALM [21, 23] and TOFHLA [22] were administered, patients with limited HL reported a tremendous amount of shame in sharing their results with their health care providers. However, VanGeest and colleagues [24] found that among those completing the NVS, nearly all patients—regardless of NVS score—reported that screening did not cause them to feel shameful and recommended HL screening in routine clinical practice.

Brief one sentence HL and numeracy screening items have been developed and tested, against various formal HL assessment tools, to quickly assess patients’ HL and numeracy skills in busy clinical settings [25–28]. While brief HL and numeracy screening items have shown promise in predicting English-speaking adults’ HL and numeracy abilities [25–28], less is known regarding the utility of equivalent Spanish-language HL screening items. For instance, Sarkar and colleagues found established HL screening items to be effective in discriminating between those with adequate versus inadequate/marginal HL, as measured by TOHLHA (administered in both English and Spanish) scores, in samples of both English and Spanish speaking diabetes patients aged ≥55 years in San Francisco, California [29]. Similarly, Cordasco et al. [30] found an established HL screening item (“How confident are you filling out medical forms by yourself?” [25]) and self-reported educational attainment of less than 6 years to be predictive of S-(Spanish)TOFHLA scores among elderly (≥65 years of age), monolingual diabetic Spanish speakers residing in Los Angeles, California.

Since Spanish speakers tend to be at greater risk for limited HL and numeracy [1], a need exists to further test the predictive value of HL and numeracy screening items across various, diverse populations throughout the United States. To our knowledge, just two studies, conducted by Sarkar et al. [29] and Cordasco et al. [30], have explored use of HL screening items in predicting TOFHLA scores among Spanish speakers. While both these studies [29, 30] provide evidence as to the usefulness of HL screening items among Spanish speakers, their study populations were limited to older (≥55 years of age) adults with established chronic disease (type 2 diabetes) residing in California. Therefore, to address gap in the literature, the purpose of this study was to evaluate the utility of brief HL and numeracy screening items in predicting NVS and SAHL scores among a sample of Spanish-speaking adults residing in a large, Midwestern city in the United States.

## Methods

### Study design and recruitment procedures

This study employed a cross-sectional design where a convenience sample of subjects completed a structured one-on-one interview, in Spanish, with a trained, bilingual (fluent in both English and Spanish) research assistant (RA). During the summers of 2012 and 2013, a total of 151 native Spanish-speaking adults from two community sites in Columbus, Ohio, United States participated in this study. The first community site was a primary care clinic that provided free of charge medical care to Spanish speaking patients. The second community site was a classroom setting where Spanish speaking adults were offered English as-a-second language instruction. In accordance to the Helsinki Declaration, The Ohio State University Biomedical Institutional Review Board approved all research and informed consent procedures employed in this study.

Depending upon community site visited, a RA approached potential study participants in either the waiting area of the primary care clinic or a small meeting room at the English as-a-second language facility. The RA explained the purpose of the study, informed the participant that his/her responses would be anonymous, and that he/she would receive a $5 supermarket gift card for partaking in the study. To be eligible to partake in the study, participants had to be ≥ 18 years of age and speak Spanish as their first (primary) language. Potential participants who appeared acutely ill, had diminished decision-making capacity, and/or had poor visual acuity were excluded. Both RAs were medical students and made the determination as to whether a participant was well enough to partake in the study. However, just one participant, over the course of both summers, was deemed too acutely ill to partake in the study. At all sites combined, approximately ≈ 86 % of adults approached to partake in the study agreed to do so. The most commonly cited reason for refusing to participate in the study was lack of interest.

### Structured interview process and instrumentation

Those agreeing to participate in the study were taken to a private area (i.e., empty clinical examination or classroom) to complete a 10 to 15 min one-on-one interview. Upon receiving verbal consent from each participant, the RA began the one-on-one structured interview in Spanish. To begin the interview, the RA administered 5 sociodemographic items (sex, age, formal educational attainment, and self-reported health status) from the 2010 Spanish-language version of the Center for Disease Control and Prevention’s Behavioral Risk Factor Surveillance Survey [31].

Second, the RA administered 3 HL and 3 numeracy screening items to each participant. The HL screening items and response options were initially developed and validated in English by Chew and colleagues [25] and then translated into Spanish by Sarkar et al. [29]. As shown in Table 1, we used the Spanish version of these HL items and response options, in our study, that were developed by Sarkar et al. [29]. The numeracy screening items and response options developed by Woloshin et al. [32] and Fagerlin et al. [33] were translated from English to Spanish for our study. Specifically, the second author and along with a native Spanish speaker collectively translated the numeracy items and corresponding response options into Spanish.

**Table 1:** English to spanish language translation of established health literacy and numeracy screening items and response options

English Language	Spanish Language
How often do you have problems learning about your medical condition because of difficulty understanding written information?^17^	¿Qué tan seguido tiene problemas aprendiendo sobre su condición médica porque es difícil entender información escrita?
*Always – Often – Occasionally – Sometimes – Never*	*Siempre – Con mucha frecuencia – A veces – De vez en cuando – Nunca*
How confident are you filling out medical forms by yourself? ^17^	¿Qué tan seguro(a) se siente al llenar formas usted solo(a)?
*Extremely – Quite a bit – Somewhat – A little bit – Not at all*	*Completamente seguro(a) – Bastante seguro(a) – Algo seguro(a) – Un poco seguro(a) – Para nada*
How often do you have someone help you read hospital materials? ^17^	¿Qué tan seguido tiene usted, un familiar, un amigo, un empleado del hospital o la clínica u otra persona que le ayude a leer materiales del hospital?
*Always – Often – Occasionally – Sometimes – Never*	*Siempre – Con mucha frecuencia – A veces – De vez en cuando – Nunca*
In general, how easy or hard do you find it to understand medical statistics?^25^	En general, ¿qué tan fácil o difícil es para usted entender estadísticas sobre temas médicos?
*Very easy – Easy – Hard – Very hard*	*Muy fácil – Fácil – Difícil – Muy difícil*
How much do you agree or disagree with the following statement? In general, I feel uncomfortable with health information that has a lot of numbers and statistics.^26^	¿Cuánto está usted de acuerdo o en desacuerdo con la siguiente afirmación? En general, no encuentro clara la información sobre salud cuando tiene muchos números y estadísticas.
*Strongly agree – Somewhat agree – Somewhat disagree – Strongly disagree*	*Totalmente de acuerdo – Algo de acuerdo – Algo en desacuerdo – Totalmente en desacuerdo*
How much do you agree or disagree with the following statement? In general, I depend on numbers and statistics to help me make decisions about my health. ^26^	¿Cuánto está usted de acuerdo o en desacuerdo con la siguiente afirmación? En general, me baso en números y estadísticas para tomar decisiones acerca del mi salud*.*
*Strongly agree – Somewhat agree – Somewhat disagree – Strongly disagree*	*Totalmente de acuerdo – Algo de acuerdo – Algo en desacuerdo – Totalmente en desacuerdo*

Third, the RA administered the 18-item SAHL [15] to each participant. The SAHL is a validated HL tool assessing Spanish-speaking adults’ ability to read and comprehend basic medical terms. Individual SAHL items are presented to the interviewee in flashcard format. The “stem” is printed at the top of the card in boldface while two associated words—“key” and “distractor”—appear at the bottom of the card. The participant is asked to identify which word is associated with the “stem” word. As an example, one card presented had the stem word “pregnancy” [*embarazo*] with the two associated words “birth” [*parto*] and “childhood” [*niñez*]. Each participant then had to distinguish whether “birth” or “childhood” was more closely related to the stem word “pregnancy.” Per established SAHL scoring guidelines, those scoring ≤14 were categorized as having inadequate HL.

To conclude the interview, the RA administered the NVS to each participant. The, NVS, consisting of 6 questions, includes a combination of reading comprehension and manipulation of numerical data to interpret content presented within an ice cream container nutrition label [16]. The valid and reliable Spanish language version of the NVS, including questions and the accompanying ice cream nutrition label, were presented to participants. As an example, one NVS question asks, “If you eat all the ice cream in the container, how many calories will you have consumed,?” [“*Si usted se come todo el helado en el envase, ¿cuántas calorías habrá consumido?”*] requiring participants to locate nutritional information on the label and apply basic math skills. Based on established NVS scoring thresholds, participants were classified as having limited (NVS = 0–1), marginal (NVS = 2–3), or adequate (NVS = 4–6) HL/numeracy skills. The NVS has been validated in previous studies [34–36].

### Statistical analysis

Data were analyzed using the Statistical Package for the Social Sciences (SPSS+ 20.0, Chicago, IL). *A priori*, statistical significance was set at *p* < 0.05. Descriptive statistics (means, standard deviations, frequencies, percentages) were conducted to depict sociodemographic and HL and numeracy characteristics of the study sample.

Spearman’s rank correlation coefficient was used to estimate the relationship between NVS and SAHL scores. Next, using both NVS and SAHL scores as reference standards, we computed area under the ROC (AUROC) curves for individual HL and numeracy screening items. AUROCs allowed us to compare the predictive ability of individual screening questions in estimating limited/marginal NVS scores or inadequate SAHL scores. An ideal question has an AUROC of 1.0, while an AUROC ≤0.5 indicates a screening item that provides no useful information.

## Results

**Fig. 1:**
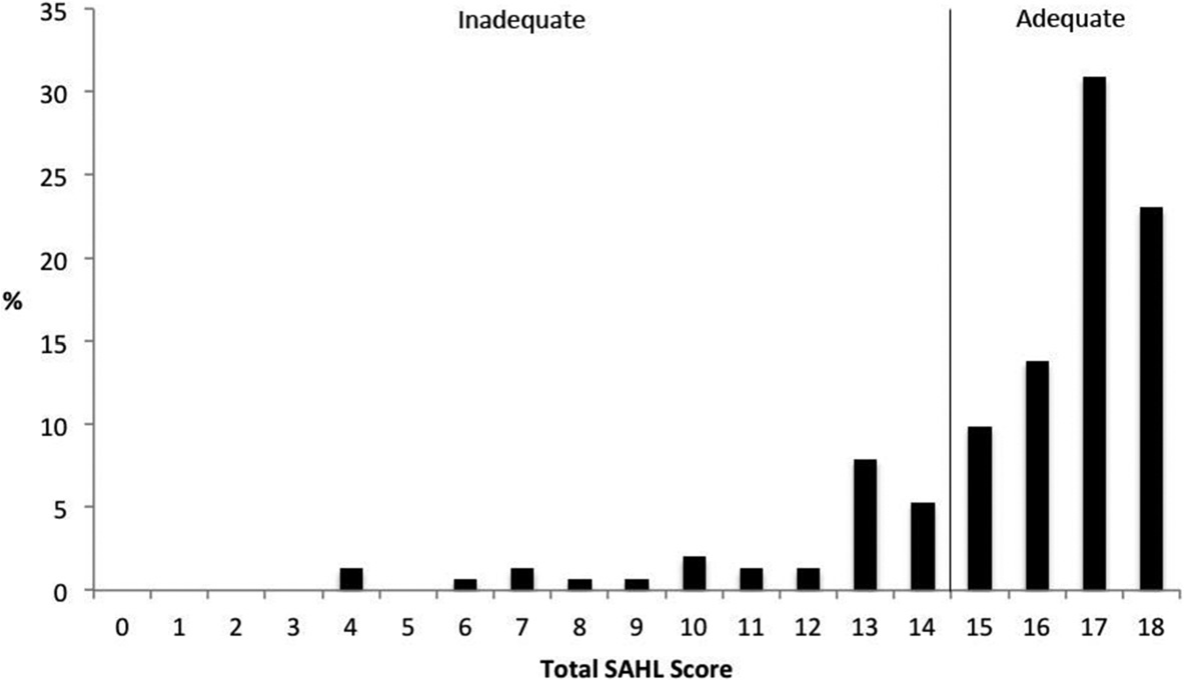
Distribution of participants’ Short Assessment of Health Literacy (SAHL) scores across established scoring thresholds

**Fig. 2:**
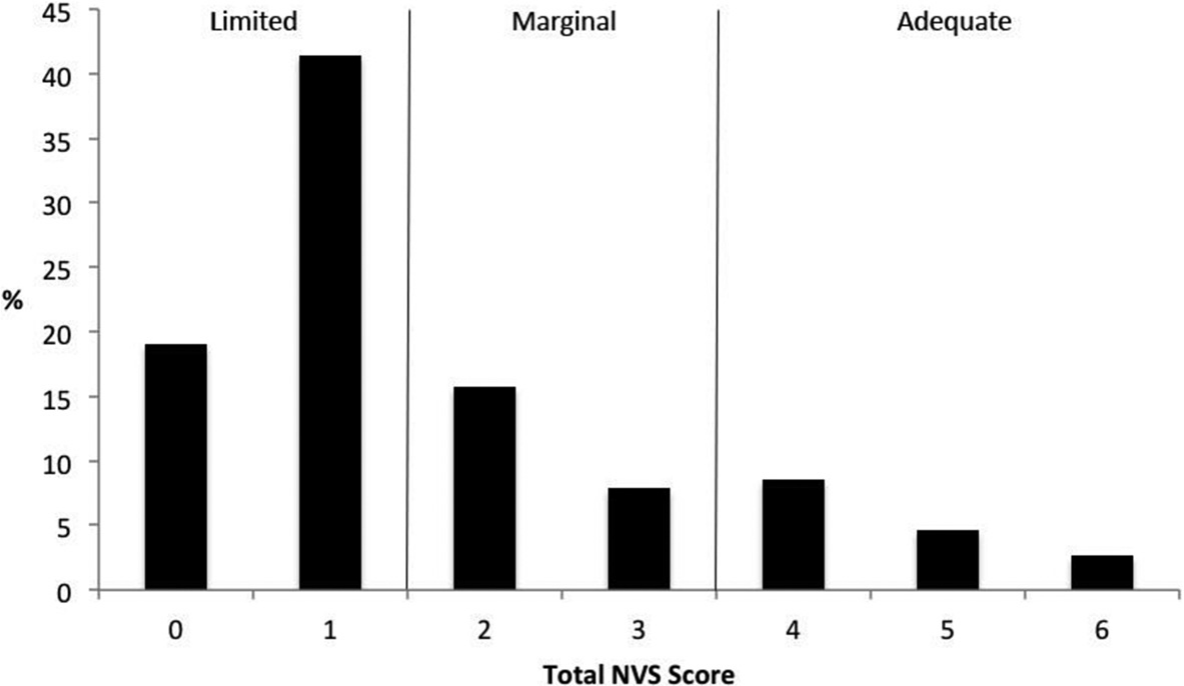
Distribution of Participants’ Newest Vital Sign (NVS) scores across established scoring thresholds

The overall sociodemographic characteristics of our sample are described in Table 2. Distribution of SAHL and NVS scores is presented in Figs. 1 and 2, respectively. Overall, participants averaged 15.7 ± 2.8 correct responses on the SAHL with 22.4 % classified as having inadequate HL (SAHL ≤14). Participants averaged 1.70 ± 1.54 correct NVS responses with the majority (60.5 %) classified as having limited numeracy (NVS score = 0 or 1). Overall, NVS and SAHL scores were weakly correlated with one another (*rho* = 0.39; *P* < 0.01) [37].

**Table 2:** Sociodemographic characteristics of study participants (*n* = 151)

Sociodemographic Characteristics	Mean ± Standard Deviation or Frequency	Percent (%)
Age in Years	36.8 ± 11.0	--
Sex		
Male	69	45.7
Female	82	54.3
Educational Attainment		
< High School	124	82.2
≥ High School	27	17.8
Self-rated Health Status		
Poor/Fair	26	17.2
Good	48	31.8
Very Good/Excellent	77	51.0

AUROCs for individual HL and numeracy screening items in identifying participants with limited/marginal NVS scores and/or inadequate SAHL scores are shown in Table 3. Across both reference standards, the HL screening item, “How confident are you filling out medical forms by yourself?”, performed best in detecting limited NVS scores (AUROC = 0.75; 95 % *CI* = 0.65–0.84), limited/marginal NVS scores (AUROC = 0.66; 95 % confidence interval [CI] = 0.57–0.75), and inadequate SAHL scores (AUROC = 0.69; 95 % *CI* = 0.58–0.79). The numeracy screening item, “In general, how easy or hard do you find it to understand medical statistics,?” was best in detecting limited NVS scores (AUROC = 0.71; 95 % *CI* = 0.60–0.83) but only moderately well in detecting limited/marginal NVS scores (AUROC = 0.61; 95 % *CI* = 0.51–0.70) and inadequate SAHL scores (AUROC = 0.59; 95 % *CI* = 0.48–0.70). For the remaining HL and numeracy screening items, AUROC scores ranged from 0.27 (“How often do you have problems learning about your medical condition because of difficulty understanding written information?”, in detecting limited numeracy) to 0.50 (“How often do you have someone help you read hospital materials”, in detecting inadequate HL).

**Table 3:** Area Under the Receiving Operating Curves (AUROC) for Health Literacy (HL) and Numeracy Screening Items Using Newest Vital Sign (NVS) and Short Assessment of Health Literacy (SAHL) standards

Health Literacy and Numeracy Screening Item	Limited Numeracy (NVS)	Limited/Marginal Numeracy (NVS)	Inadequate Health Literacy (SAHL)
How often do you have problems learning about your medical condition because of difficulty understanding written information?	0.27 (0.16–0.37)	0.46 (0.37–0.56)	0.47 (0.36–0.56)
How confident are you filling out medical forms by yourself?	0.75 (0.65–0.84)	0.66 (0.57–0.75)	0.69 (0.58–0.79)
How often do you have someone help you read hospital materials?	0.42 (0.27–0.57)	0.47 (0.37–0.56)	0.50 (0.40–0.60)
In general, how easy or hard do you find it to understand medical statistics?	0.71 (0.60–0.83)	0.61 (0.51–0.70)	0.59 (0.48–0.70)
How much do you agree or disagree with the following statement? In general, I feel uncomfortable with health information that has a lot of numbers and statistics.	0.44 (0.32–0.57)	0.49 (0.40–0.59)	0.47 (0.36–0.59)
How much do you agree or disagree with the following statement? In general, I depend on numbers and statistics to help me make decisions about my health.	0.44 (0.32–0.57)	0.49 (0.39–0.58)	0.40 (0.30–0.50)

Numeracy and HL levels based on NVS scores (limited = 0 or 1; marginal = 2 or 3) and SAHL scores (inadequate ≤14). AUROCs calculated with 95 % confidence intervals

## Discussion

This study examined utility of brief HL and numeracy screening items in predicting NVS and SAHL scores among a sample of Spanish-speaking adults residing in a large, Midwestern city in the United States. Overall, participants in this study had lower numeracy as compared to general HL skills as measured by the NVS and SAHL, respectfully. The most important finding from our study was that two screening items (“How confident are you filling out forms by yourself?” and “In general, how easy or hard do you find it to understand medical statistics?”) were both strong predictors of overall NVS and SAHL scores.

Our findings extend the results of prior studies in which the “confident with forms” item was the best predictor of identifying patients at risk of inadequate HL [25–30, 38]. In contrast to Sarkar et al. [29], who found that all of Chew’s [25] screening items performed well in Spanish speakers, the “confident with forms” item was a much better predictor of NVS and SAHL scores in our study population. However, our results mirror those of Cordasco et al. [30], who found the “confident with forms” item to be the best predictor of inadequate TOFHLA scores in their population of monolingual Spanish speakers in Los Angeles, California. The combined findings of our study and Sarkar’s, and Cordasco’s studies provide strong evidence for the benefit of using a single Spanish HL screening item to estimate a participants’ HL and/or numeracy skills.

In comparison to earlier studies in English-speaking populations [25–30, 38], Chew’s [25] brief HL screening items had lower AUROCs among our Spanish-speaking population. In part, this may be due to participants’ interpretation of the items as referring to forms and materials written in English, not Spanish. Thus, participant responses may reflect difficulties in reading and interpreting materials in English, rather than their ability to read and respond to materials written in their native language. Future work, could perhaps, specify that written material and forms refer to those in the participant’s native (Spanish) language.

Unlike Sarkar et al. [29] and Cordasco et al. [30] who used the TOFHLA as their reference standard, we used the NVS and SAHL as reference standards. It is interesting to note that while nearly 60.5 % of our Spanish speaking population had limited HL (using SAHL scores as the reference) and 84.2 % had limited/marginal based on NVS scores, only 22.4 % were categorized as having inadequate HL based on SAHL scores. Our findings are similar to those reported by Ramirez-Zohfeld and colleagues [39] in that categorization of participants into levels of literacy is likely to vary across HL assessment tool employed.

The overall proportion of low NVS scores could reflect a combination of both low numeracy-related skills and/or unfamiliarity with nutrition labels among Spanish speakers. Many of the participants tested were unsure of how to read and interpret the ice cream nutrition label provided. Thus, NVS scores may not provide good discrimination of numeracy skill levels among Spanish speakers. Future investigations should examine and compare the validity of different HL/numeracy assessment tools in Spanish-speaking and other diverse populations. Additionally, research is needed to explore whether gender differences exist in self-report of health literacy. For example, among a large sample of Taiwanese adults, Lee et al. [40] found that women’s responses to HL screening items reflected their actual HL scores (as assessed by the Mandarin Health Literacy Scale), whereas men tended to over-report their actual HL skills. Further study is warranted to see if these findings emerge in Spanish-speaking populations as well.

Our study findings should be considered in light of several potential limitations. First, participants in this study were sampled in a single Midwestern city in the United States drawn largely from free clinics where patients are often uninsured and/or have modest incomes. As a result, our findings may not be representative of other more geographically and/or socioeconomically diverse settings. Second, we were unable to stratify participants into more detailed categories of educational attainment due to differences in schooling and descriptions used to describe the educational systems in Spanish-speaking countries. Third, our findings reflect the criterion validity of the HL and numeracy screening items against both the NVS and SAHL. Fourth, as with all observational studies that rely on self-reports, response bias remains a possibility.

## Conclusions

Brief one sentence HL and numeracy screening items have utility in screening for limited HL and numeracy in Spanish-speaking adults. The “confident with forms” item discriminates well amongst Spanish-speaking adults with varying HL and numeracy skills. This single question can be easily administered in a variety of clinical settings and eliminate the need for more formal and lengthy HL and/or numeracy assessments.
